# A Qualitative Evaluation of the Implementation of an Intimate Partner Violence Education Program in Fracture Clinics

**DOI:** 10.1007/s10896-019-00052-4

**Published:** 2019-05-11

**Authors:** Sheila Sprague, The EDUCATE Investigators

**Affiliations:** Department of Surgery, McMaster University, 293 Wellington St. N., Suite 110, Hamilton, ON L8L 8E7 Canada

**Keywords:** Intimate partner violence, Domestic violence, Family violence, Medical education, Qualitative methodology

## Abstract

We developed an intimate partner violence educational program (EDUCATE) for health care providers which was implemented in seven fracture clinics by local IPV champions. The purpose of the program was to provide health care providers with the knowledge and skills required to comfortably identify and assist women experiencing IPV in the fracture clinic. The program consisted of an introductory video, interactive online modules, and an in-person presentation by a local IPV champion. The study aim was to qualitatively evaluate the feasibility, acceptability, and perceived value of the program. We conducted semi-structured interviews with 10 champions and 23 participants and identified themes using a qualitative descriptive approach. Champions and participants expressed a strong satisfaction with the program. Champions also described several barriers and facilitators to program implementation. Additionally, we identified themes through analysis of interview data from champions (champion training, program delivery, and perceptions about program participants’ receptiveness to the training) and participants (value of the training experience, useful program content, desire for more education, and suggested program improvements). The program showed promising results, as both champions and program participants had overall positive experiences completing the program. Their suggestions for improvement have been used to refine the program, which is now publically available for educational purposes through www.IPVeducate.com.

## Introduction

Intimate partner violence (IPV), sometimes referred to as domestic violence, is defined as harm inflicted by one’s past or current partner and may consist of physical, sexual, economic or psychological abuse (World Health Organization 2005). Globally, one in every three women who has ever been in a relationship has experienced IPV at some point in her life (World Health Organization, London School of Hygiene and Tropical Medicine,, and South African Medical Research Council 2013). IPV is associated with many negative physical and mental health consequences for victims (Centers for Disease Control and Prevention 2008; Coker et al. 2002; Ellsberg et al. 2008) and thus an increase in health care resource utilization (Gass et al. 2010; Rivara et al. 2007). In the US, the annual costs associated with IPV exceed 8.3 billion dollars (Centers for Disease Control and Prevention 2017). IPV is also a significant risk factor for intimate partner homicide, with 38% of all female homicides committed by intimate partners (World Health Organization et al. 2013). Previous research has found that 45% of these intimate partner homicide victims presented to health care providers (HCPs) within the 2 years preceding their death seeking treatment for an injury sustained through IPV (Rivielo 2010).

IPV is therefore a relevant topic to all HCPs; however, it is specifically relevant to orthopaedic practice. Previous research has identified musculoskeletal injuries (i.e. sprains, fractures, and dislocations) as the second most common physical manifestation of IPV (Bhandari et al. 2006). Furthermore, a large prevalence study found that one in six women presenting to fracture clinics has experienced IPV in the past year (PRAISE Investigators et al. 2013). Orthopaedic surgeons and other HCPs treating women in fracture clinics are therefore uniquely positioned to identify IPV and provide critical assistance by initiating referrals to services such as shelters, counsellors, or support groups. However, HCPs often report a lack of confidence in asking about IPV and assisting patients who are experiencing it (Bhandari et al. 2008; Shearer and Bhandari 2008; Shearer et al. 2006). Recent research suggests that these challenges can be overcome with educational programs within clinical settings (Sprague et al. 2017).

To address this need, we developed an IPV educational program (EDUCATE) specifically for orthopaedic surgeons, surgical trainees, and other HCPs who care for patients in a fracture clinic setting (EDUCATE Investigators 2018a, b). The purpose of EDUCATE is to provide participants with the knowledge and skills required to comfortably identify and assist women experiencing IPV. Since the implementation of the EDUCATE program, a multicentre quantitative and qualitative evaluation have been performed. The quantitative study results have been published elsewhere (EDUCATE Investigators 2018a, b). The qualitative study focussed on the feasibility, acceptability, and perceived value of the program explored through the experiences of local champions and program participants. The results of this study were used to improve and enhance the EDUCATE program prior to widespread implementation. The revised version of the program is publically available for educational purposes and can be accessed at www.IPVeducate.com.

## Methods

**Fig. 1:**
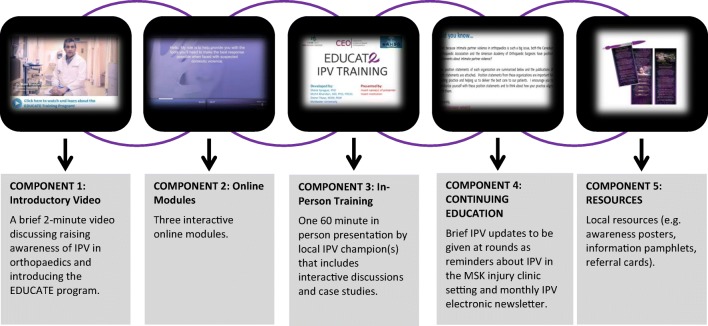
EDUCATE program components

The EDUCATE program was comprised of three components (see Fig. 1). The first component was a short introductory video that introduced the EDUCATE program and discussed the importance of IPV identification and assistance within fracture clinics. The second component included three interactive online modules from the “Responding to Domestic Violence in Clinical Settings” training series (Mason and Schwartz 2018). The final component was an in-person training session (approximately 1 h in length) on how to identify and assist women experiencing IPV within the fracture clinic. This training session included slides, video demonstrations, role playing, case studies, interactive discussions, and information about hospital and community resources. The EDUCATE program took approximately 2 h to complete. Following completion of the program, short bi-monthly training updates were distributed electronically to program participants. These updates provided information to enhance IPV knowledge and encourage HCPs to continue practicing EDUCATE principles.

The EDUCATE program builds upon a previous educational program developed and evaluated by members of the study team (Madden et al. 2015). The EDUCATE program was based upon Bandura’s self-efficacy theory for changing behaviour (Bandura 1977). As current evidence indicates that a multi-faceted approach results in a higher uptake and retention of knowledge (Gadomski et al. 2001), the EDUCATE program included multiple training methods as described above. The program also incorporated adult learning principles, which is the hallmark of problem-based learning (Wood 2008). Problem solving is a central component of self-management and is a key element of most successful individual and group self-management programs reporting improved outcomes (Funnell 2010). Research on both adult education and effective knowledge transfer suggests that interactive strategies are necessary to be successful (Harris et al. 2002; Lavis et al. 2003; Short et al. 2006b; Zaher et al. 2014). To develop the EDUCATE program, we conducted a scoping review which reviewed and synthesized all of the literature evaluating IPV educational programs in health care settings (Sprague et al. 2017). Additionally, we held in-depth consultations with orthopaedic surgeons and a social worker with over 25 years’ experience working with IPV victims. Finally, drafts of the program were reviewed by members of our knowledge user team which consisted of representatives from the Canadian Orthopaedic Association, family medicine, emergency medicine, physiotherapy, midwifery, and IPV services.

The EDUCATE program recognized that IPV can impact women of all different backgrounds and advocated for routine IPV inquiry for all female fracture clinic patients, regardless of age, race, relationship status, and socioeconomic status. While both women and men can be victims of IPV, women bear the majority of harm from IPV as they experience it more frequently and severely than men (Breiding et al. 2014; Roberts et al. 1996; Statistics Canada 2013). Because of this, IPV is recognized by leading health and humanitarian authorities as a gender-based problem and violation of women’s human rights (United Nations 1994; World Health Organization et al. 2013). Consequently, the EDUCATE program was focussed on teaching HCPs to identify female patients who are experiencing IPV and providing critical assistance by responding to IPV disclosures in a supportive manner, assessing immediate safety, and initiating referrals to community services such as shelters, IPV counsellors, and support groups.

The EDUCATE program used a “train-the-trainer” approach, whereby one or more individuals from participating fracture clinics (i.e. surgeons, surgical trainees, non-physician HCPs, or research coordinators) were asked to become local EDUCATE champions. Local IPV champions received in-depth training about the EDUCATE program from a social worker (DT). The champion training was delivered through a 1.5 h in-person session held at a large annual meeting of a prominent orthopaedic association. Prior to the training, champions reviewed all components of the EDUCATE program (i.e. watched the introductory video, completed the interactive online modules, and reviewed the slide deck for the in-person training presentation). During the training session, the social worker reviewed the in-person training presentation with champions and elaborated on key concepts and teaching points. The champions were then provided with an opportunity to ask questions. The meeting was attended by seven of the eleven champions from four of the seven participating fracture clinics. The remaining four champions from three of the participating fracture clinics received the same training via teleconference. Local IPV champions were responsible for becoming program curriculum experts to implement the program at their local fracture clinics and were encouraged to tailor the training content to maximize applicability.

The EDUCATE program was implemented at seven North American fracture clinics, six in Canada and one in the United States beginning in November 2016. All EDUCATE champions received the champion training prior to implementing the program. All local orthopaedic surgeons, surgical trainees, and other HCPs who treat patients at participating fracture clinics were then invited by their local champions to participate in the EDUCATE program. The number of potential participants across each site varied from 12 to 86 (Hamilton Health Sciences – General Site = 39, University of Calgary = 49, Memorial University of Newfoundland = 32, St. Michael’s Hospital = 28, London Health Sciences Centre = 37, St. Joseph’s Healthcare Hamilton = 12, and the CORE Institute = 86). Training and implementation at each site were completed by June 2017.

The project manager from the McMaster University Methods Centre (TS), invited all champions, via email, to participate in a qualitative interview about their experiences implementing and delivering the EDUCATE program at their respective fracture clinics. At least one champion from each site consented to participate in an interview. All champion interviews were conducted by TS, lasted approximately 20 min, and were completed between 1 and 105 days after program implementation. Champions were professionally acquainted with the interviewer and had a professional relationship with the Principal Investigator at the time of the study. Additionally, local research personnel from each fracture clinic identified a subset of program participants via purposeful and maximum variation sampling to participate in a qualitative interview about their experiences receiving the program. Purposeful sampling aims to identify information rich cases (i.e. participants who are likely to provide in-depth information about the phenomenon of interest) (Patton 2002). Maximum variation sampling aims to achieve diversity within the sample on key variables (Sandelowski 2000). Specifically, local research personnel sought out participants, via email or in-person invitations, who they thought would provide detailed information about their experiences and differed in terms of health care profession. If participants agreed to complete an interview, local research personnel connected the participant with the interviewer (TS) via email. All program participant interviews were conducted by TS, lasted approximately 20 min, and took place within zero to 256 days after program completion. Participants had no personal or professional relationship with the interviewer or Principal Investigator at the time of the study.

During semi-structured interviews, champions and participants were asked about their experiences with program implementation and participation and shared their perspectives on the feasibility, acceptability, and perceived value of the program. The interviewer used reflection, paraphrasing, clarifying and summarizing techniques in all interviews to provide interviewees with the opportunity to clarify and add new ideas to the discussion. Interviews were conducted over the phone or in-person with an interview guide comprised of semi-structured questions to inform the discussion and allow for exploration of new topics. No one else besides the interviewer and participant was present for the interviews. All interviews were audio recorded with participants’ consent and transcribed verbatim. Interview invitations continued to be sent until data saturation was achieved. We considered the data to be saturated when no new themes emerged, and we ad obtained interview data from at least one participant at each of the participating sites (excluding the site in which no participants agreed to participate in an interview).

Data collection was a reflexive and interactive process where data collection and analysis occurred simultaneously to allow new themes identified in early interviews to be explored in subsequent interviews, and to monitor for data saturation. All interview transcripts were coded by two of three independent reviewers and analysed using a conventional qualitative content analysis (Hsieh and Shannon 2005; Sandelowski 2000). This approach was used to provide a descriptive summary of the data using participant’s own language with minimal theoretical interpretation (Sandelowski 2000).

This study was approved by the Research Ethics Board at McMaster University (REB# 1816) as well as the Research Ethics Boards or Institutional Review Boards of each participating site and was conducted in accordance with humane and ethical research principles.

## Results

Ten champions and 23 program participants consented to participate in the qualitative interview process. Aside from one site in which no participants consented to an interview, the minimum number of participants from each site was 1 and the maximum was 9 with a mean of 3. EDUCATE champions were predominantly orthopaedic surgeons, however participants also included non-physician HCPs, and orthopaedic surgery residents (see Table 1). The mean age of participants was 41.5 (standard deviation (SD): 11.8). Fifty-seven percent of participants were male and most were Caucasian (87.0%). Participants had been in practice for a median of 5 years (1st quartile: 3; 3rd quartile: 28) at the time of training.

**Table 1:** Program participant demographics

Variable	Statistics *n* = 23
Age, years: mean (SD)	41.5 (11.8)
Gender: count	
Male	13
Female	10
Ethnicity: count	
Caucasian	20
South Asian	2
Asian	1
Health care profession: count	
Orthopaedic surgeon	8
Surgical trainee	7
Nurse	3
Orthopaedic technician	3
Physician assistant	1
Physiotherapist	1
Number of years in practice: median (1st and 3rd quartiles)	5 (3, 28)
Previous IPV training: count	19

*SD* standard deviation, *IPV* intimate partner violence

### Perceived Value of the Program

Both champions and EDUCATE program participants reported high satisfaction with the IPV education program. This included champions, “I think that everyone took away something [from the program’]”, more experienced providers, “it’s a necessary, valuable program”, and more novice providers, “I thought it was a really helpful program, especially being in my first year of residency, I felt it was nice to kind of have that validational baseline knowledge”. Participants discussed plans to incorporate the training they received from the program into their practice through increased IPV screening. One participant stated, “I’ll be more likely to ask certain questions with my awareness being raised”. Another participant expressed, “I think that [IPV is] something that I’ll look for in future residency and during practice as well”. One participant also shared an example of a change in screening practice due to the EDUCATE program, “There was a patient that I saw a couple weeks back where I did screen for IPV, and I would not have before the training...”. Similarly, one champion shared that, “I’ve already had people come up to me and say, this person said x, y, and, z you know, where are those [IPV] information sheets.” The experiences of both champions and participants, though unique, both demonstrated that the program was perceived to be valuable.

### Feasibility of Program Implementation

When asked about the implementation of the EDUCATE program, champions identified a series of barriers and facilitators.

#### Barriers

Champions described time constraints, ensuring completion of the online training modules, and redundancy in content, as barriers to program implementation. Champions discussed difficulty identifying time to schedule training, both in their own schedules and with the schedules of participants. Champions suggested that for the program to be sustainable, it would need to fit into existing educational times such as webinars or symposiums through professional orthopaedic associations, or use of site-specific training videos or online modules, and incorporation into residency curricula. For instance, one champion expressed it was easier when “we just made this part of our educational curriculum”. Champions also expressed difficulty in ensuring completion of the online training modules since the modules had no built-in way of tracking who had completed the training. Champions from two sites chose to require completion of the online modules by providing them in a group setting; however, this increased the time commitment. Lastly it was stated that some of the content from the online modules was repeated in the in-person presentation, increasing redundancy. This redundancy, although identified as a barrier, was viewed as a potential benefit by some champions to underscore key points.

#### Facilitators

Delivering training during protected academic or professional development time, obtaining buy-in from key personnel, and inclusive training were identified as facilitators to program implementation. As finding time for EDUCATE training was identified as a barrier, it is not surprising that champions identified using protected academic or professional development time to deliver the program as a potential facilitator. Doing so made the EDUCATE program more feasible for participants as “the expectation is that as many providers as possible attend academic day”. In addition, obtaining buy-in from key administrators was a crucial step in securing protected academic or professional development time for training and it encouraged others to view the training as important which, in turn, encouraged attendance. Similarly, champions reported having inclusive training as a facilitator. Champions described an added value in having as many people from the fracture clinic as possible participate in the training. This allowed participants to learn from others with more experience and become comfortable integrating the knowledge gained training into their practice because “everybody was at the teaching session and knows that this is something that we should be doing”.

### Feasibility of Program Delivery

Following a discussion on implementation of the program, champions were asked about their experiences delivering the EDUCATE program. Three themes were identified; champion training, program delivery, and perceptions about program participants’ receptiveness to training. Champions expressed satisfaction with the champion training they received prior to implementing the program at their respective fracture clinics “I thought it was very helpful, [the champion trainer] did a nice job, in giving examples, explaining things further, and really emphasizing key points. So, I thought that was extremely helpful”. Champions expressed that it helped them to become familiar with the program content which allowed them to overcome challenges that arose in training. Champions who received training via teleconference expressed similar satisfaction with the training and did not share differing experiences from champions who attended the in-person champion training session. There were several comments however stating that there may have been too long of a gap between the champion training and implementation at the fracture clinic,

…the only thing was that I thought I did it too far in advance of when we actually trained staff here, because I found myself going back through the presentation and trying to remember what I was actually supposed to teach them.

Most champions reported feeling comfortable in delivering the program, “I felt quite comfortable with it, with the preparation that I had from [the champion training]” but stressed the importance of customizing the program to include their local resources “…we really liked having a bit of freedom with the presentation at the end because you could really tailor it to…our local resources and what was expected of [the residents] at the clinical site that they worked at”. Champions also discussed the benefit of adapting the program to include cases from their own practice rather than generic case studies. Many preferred this approach because they could present the case in greater detail, show corresponding x-rays, and share patient outcomes. Lastly, champions often commented on the perceived receptiveness of program participants to the training. Champions indicated that overall, they thought that participants appreciated receiving the EDUCATE program, particularly the in-person training component, and found it to be a valuable experience. Champions reported that participants were engaged and receptive throughout the program and,

If they had any questions they weren’t shy to ask them, and from the questions they were asking me, it seemed like they were taking information, processing it, and then thinking…beyond what does that mean for me and what would that actually look like in clinic.

### Acceptability of the Program

Three themes were identified from participant interviews that related to the acceptability of the program including value of the training experience, useful program content, desire for more education, and suggested program enhancements. Participants in the EDUCATE program expressed the value in an increased awareness of IPV, a need for the EDUCATE program and an appreciation of the in-person component of the training. One participant explained that,

As a health care worker…we get very minimal training in this area unless you’re actively involved with women’s health, or you know working in an assault violence workplace, we get I would say no training for this. So, for all of us I think it was very good to go attend the women’s EDUCATE seminar…

This interest in the program was also observed by champions, “I felt that I had a very engaged audience, they were very interested”. Participants discussed appreciation of the in-person component, along with other aspects of the program content and delivery, with one participant explaining,

…I think it helps to be in person, particularly with a topic like this because I actually asked our [champion] how he talks to patients about this, and has he identified patients, and what’s his experience with this?...It’s not just, understanding the background and the statistics and all that kind of the thing, but actually how do you approach this sensitive topic in a real way…

The appreciation of the in-person training session was also observed by champions with one champion noting, “I think that people appreciated somebody actually taking the time to go through the strategies for an issue that I know all the staff think is important…”. While attributing value to the in-person training component, a few participants expressed that there was some repetition between the online modules and the in-person presentation; however, similar to the champion reports, views differed as to whether this repetition was beneficial. Additionally, though participants reported sufficient detail and length of the program, they would have appreciated more practical guidance on how learning can be incorporated into clinical practice “if [the champions] could provide… a video of them role playing or [could] list some of the phrases they commonly use and what they found that works well in their practice”. Based on participant feedback the EDUCATE program, although containing information on local resources, could have included additional information on practical applications and further education in IPV. For instance, participants expressed some remaining uncertainty about where to locate IPV resources within their fracture clinics “…if there was anything to stress it was ‘okay, exactly where are these resources’ because at some point it wasn’t exactly clear where in the clinic I’d find them”. Participants also expressed wanting to know more about reporting requirements for IPV when children are involved, specific phrases to use when asking about IPV, residents’ roles in the process of initiating IPV assistance, and victim follow-up obligations. Participants suggested several program enhancements to address some of these uncertainties. These suggested enhancements included videos of IPV champions demonstrating how to ask and assist with IPV in the fracture clinic setting, more discussion of local IPV cases, opportunities for small group discussions during training, monthly meetings to practice and update skills, and additional information on legislative IPV reporting requirements.

## Discussion

Our study qualitatively evaluated the experiences of local champions and program participants throughout the implementation and delivery of an IPV educational program specifically for fracture clinics. The results of this study have been used to improve the EDUCATE program, and a revised version of the program is now available to fracture clinics across Canada. We found that overall, program implementation was a positive experience for both local champions and participants, and that the program was perceived as valuable, acceptable, and feasible. Participants felt the program increased their awareness of IPV and that the training was relevant to their practice. This is inline with the results from the quantitative evaluation of the EDUCATE program which found that participants’ IPV-related knowledge, attitudes, beliefs, and behaviours, as measured by the Physician Readiness to Manage IPV questionnaire (Short et al. 2006a), significantly improved after completing the program (EDUCATE Investigators 2018a, b).

Overall, EDUCATE program champions felt implementing and delivering the program in their fracture clinics was feasible, and expressed positive experiences. To the best of our knowledge, no previous studies have looked at the experiences of individuals who deliver IPV educational programs within health care settings. However, other studies have employed a “train-the-trainer” approach with positive results (Bonds et al. 2006; Gadomski et al. 2001). Specifically, this research found that educational programs delivered by local IPV experts who received training, allowed practices to tailor the intervention to best fit their needs and led to increases in IPV screening (Bonds et al. 2006) and improvements in HCP self-efficacy (Gadomski et al. 2001). In our study, champions identified time constraints, ensuring completion of the online training modules, and redundancy of information as barriers to program implementation and using protected academic or professional development time, obtaining buy-in from key personnel, and inclusive training as facilitators. Some similar barriers and facilitators were identified by Campbell et al. in a process evaluation of an IPV educational program (Campbell et al. 2001). Specifically, local trainers identified time and money as barriers to providing ongoing training. They also identified strong support and administrative backing as important facilitators for training. This is similar to the theme of obtaining buy-in from key personnel that our study identified as a facilitator. It is also interesting to note that some of the facilitators identified by champions in our study may help to overcome the barriers of time constraints. For example, buy-in from key personnel may help potential participants to view completing all elements of the EDUCATE program as a priority when time is limited. Additionally, scheduling training during protected academic or professional development time may minimize the burden of time by allowing it to fit into already existing schedules. Based on these barriers and facilitators, we developed an implementation guide for champions that provides step-by-step instructions on how to implement the program and tips to help champions maximize efficiency. We recommend that anyone looking to implement the EDUCATE program, or other similar educational programs, in a fracture clinic setting should work closely with clinic administrators and other influential team members to ensure they understand the value of the educational initiative. Influential team members may include clinical managers, residency program coordinators, charge nurses, and chief residents. These individuals may also be able to grant permission to use protected academic or professional development time to deliver the training and also encourage other colleagues to take time out of their busy schedules to attend.

Overall, EDUCATE program participants believed that the training they received was valuable, that the program was acceptable. This viewpoint was also echoed by champions. Participants also expressed a desire for additional education and suggested enhancements to the program. This finding is in line with the results of a previous scoping review of the literature on IPV educational programs which found that multiple studies reported positive program evaluation results (Sprague et al. 2017). Additionally, many of the themes identified in our study were similar to themes identified in a qualitative study by Lo Fo Wong, Wester, Mol, and Lagro-Janssen (Lo Fo Wong et al. 2007). In this study, family physicians expressed that the IPV training they had received had increased their suspicion criteria and awareness of IPV, that they believed the training was useful in their daily practice, and that they desired additional IPV education. Participants in our study also suggested several program refinements such as the inclusion of videos of IPV champions demonstrating how to ask and assist with IPV in the fracture clinic setting, more discussion of local IPV cases, opportunities for small group discussions during training, monthly meetings to practice and update skills, and additional information on IPV reporting requirements. Based on these recommendations, we refined the EDUCATE program to include a video library showing different HCPs demonstrating how to ask about IPV and provide assistance upon disclosure. These videos provide program participants with specific phrases to use when asking about IPV, and model different ways of introducing the conversation and responding when a patient does or does not disclose IPV. Additionally, we have revised the in-person presentation to include additional information on IPV reporting requirements, including information about where HCPs can seek additional guidance for case-specific questions. Finally, we have also revised the champion training to emphasize the importance of including local IPV cases and opportunities for small group discussion. We recommend that others developing similar programs allow room to tailor the program to each individual fracture clinic to personalize the training experience for participants and maximize learning. We also recommend that training programs include practical information to help participants readily apply learning to their clinical practice. Finally, program implementation should involve more than just a single training session. It should be an ongoing initiative to ensure that participants continue to retain and enhance their knowledge gains. While champions and program participants disagreed on the value of redundancy in the training content, previous research has showed that repetition is valuable when learning new clinical skills (Bosse et al. 2015). Consequently, the redundant content was not removed from the EDUCATE program, however, the online training modules were changed to an optional component of the program for those looking for further information, or a training booster. We recommend that training programs include as much redundancy as possible to reinforce key concepts, while ensuring that the required time commitment is not overly burdensome or prohibitive.

Finally, participants expressed plans to incorporate training into their practice, with some participants already reporting changes. This accords with previous research which has found that IPV education can impact HCPs awareness of their role in IPV identification and assistance within their practice (Protheroe et al. 2004) and result in increases in IPV identification and assistance (Feder et al. 2011; Thompson et al. 2000). However, even after IPV education some HCPs may still lack confidence in IPV care that interferes with their ability to integrate training into practice (Bacchus et al. 2010). A previous systematic review was conducted to examine the ability of IPV educational programs to improve physicians’ knowledge, identification, and management of IPV (Zaher et al. 2014). This review included nine randomized controlled trials that addressed this question and found that IPV educational programs were generally effective at improving knowledge; however, only those that were combined with system support interventions resulted in improvements in IPV identification and assistance behaviours. Examples of system support interventions include; training on local IPV resources, prompts in medical records to screen for IPV, and IPV awareness posters and resources in clinics. While the EDUCATE program already included information about local IPV resources and IPV resource lists for patients, the revised program includes several IPV awareness posters that can be displayed in the fracture clinic to let patients know that the fracture clinic is a safe place to discuss IPV. Additionally, we have also created posters for staff areas of the clinic to remind HCPs to ask their patients about IPV and where IPV resources for patients are located in the fracture clinic. We recommend that others developing similar programs implement these programs in conjunction with system level changes to ensure the fracture clinic is setup in a way that complements the educational program and provides HCPs with the necessary supports to incorporate the training into their practice.

### Limitations

Due to the design, this study has limitations. Firstly, recruiting volunteers to participate likely led to the inclusion of a disproportionate number of individuals in our study who are interested in the topic of IPV and IPV education. However, since the goal of purposeful sampling is to identify and include individuals likely to provide in-depth information about their experiences participating in the educational program, this selection aligns with our study objective. It is also possible that participants who valued the program were more likely to agree to participate in an interview than participants who did not value the program; therefore, interview data we obtained may not be representative of all possible participants. Secondly, our study is limited by a small sample size in terms of participating institutions, champions, and program participants. However, small sample sizes are common in qualitative research and generalizability of findings to other populations is typically not the aim, rather the intent is for the reader to determine the transferability of results to their clinical environment (Leung 2015). Thirdly, we were not able to recruit participants from one site into the qualitative arm of the study. Unlike other sites that participated in the study, this site was located in a jurisdiction with mandatory IPV reporting for HCPs. It is possible that because of this, or other reasons, participants from this site had a different experience than participants at other sites. Fourthly, our sample was not culturally diverse in that 87% of participants were Caucasian. However, previous research has found that orthopaedic surgery lags behind other areas of medicine in terms of diversity, so it may not be surprising that our sample also lacks cultural diversity (Zuckerman 2018). Finally, the seven institutions that participated have all been previously involved in IPV research and therefore may have more knowledge and awareness of the subject than other fracture clinics.

## Conclusion

A qualitative evaluation of the implementation of the EDUCATE program found that both champions and program participants had overall positive experiences completing the program. Champions and participants suggested several strategies to improve the program that were used to refine it prior to widespread implementation.
